# Book Review

**Published:** 1932

**Authors:** 


### Reviews of Books

**An Index of Treatment.** By Various Writers. Tenth Edition. Edited by Robert Hutchison, M.D., F.R.C.P. Pp. xviii., 1,027. 93 illustrations in the text. Bristol: John Wright & Sons Ltd. 1931. 42s.—Hutchison's *Index of Treatment* is too well known to require any recommendation. The tenth edition has recently appeared, and contains many valuable additions, particularly "Diabetes in Childhood," by George Graham, although the author is, in our opinion, inclined to restrict carbohydrates too much. "Pink Disease" or "Acrodynia" is dealt with by Donald Paterson, whilst "Protein Shock" is briefly and clearly described by Sir Thomas Horder. Some articles have been rewritten, notably that on the treatment of empyema, an excellent résumé of modern surgical procedures, which only figures in the index under the heading of "Pyothorax," although in the text it is placed under the "E's." But it is sufficient to say that the new edition of Hutchison's *Index of Treatment* is as indispensable to every practitioner as the earlier editions.

**A Synopsis of Surgical Anatomy.** By Alexander Lee McGregor, M.Ch. Pp. xiv., 609. 606 Illustrations. Bristol: John Wright & Sons, Ltd. 1932. Price 17s. 6d.—This book hails from South Africa, and deserves well of us if for that reason only: it claims to be the first-born of the Medical Department of the University of the Witwatersrand. It is a formidable publication of over 600 pages, and is complete with preface, foreword and introduction. We question whether this is not tending to frighten off the student from the study of an ancillary subject, of great importance though it admittedly is, when his time is so fully occupied with clinical study. It seems to us that the tendency to overdo the pure anatomy to the exclusion of the more distinctly applied part of the subject tends to defeat the laudable object with which such books are written. No one ever wrote a more readable and valuable book on this subject than Treves, an anatomist as well as a surgeon, and he introduced many an apt clinical detail to emphasize the point he would make. It made his book an excellent bridge between anatomy and surgery, and emphasized the value of the former in a most practical way.

In this book there is a division into two almost exactly equal parts of the anatomy of the normal and of the abnormal. Both are good, the latter excellent, so much so that we should like to see the first part omitted altogether. It is practically pure anatomy, and not what a student in his clinical years wants, or will find time to read. The second section is exceedingly good, full of sound anatomy served up in an appetizing and useful form. It may be warmly commended to anyone who wants to retain what is useful in all the anatomy he has learned and to learn how to make the best use of his knowledge in acquiring clinical acumen. The very numerous illustrations are clear and well chosen and the index is adequate.

**Handbook of the Vaccine Treatment of Chronic Rheumatic Diseases.** By H. WARREN CROWE, D.M. Second Edition. Pp. ix., 79. London: Oxford University Press, 1932. Price 3s. 6d.—The treatment advocated in this handbook is based on the hypothesis (it can hardly be called more) that a strain of skin staphylococcus, named by the author Micrococcus Deformans, plays a predominant part in the production of the condition known as rheumatoid arthritis, and that various streptococci of alimentary type have an equally important share in causing fibrositis and osteo-arthritis. One main purpose of the book is to contend that an autogenous vaccine is not absolutely necessary, but that satisfactory results can usually be obtained by the use of a "stock" vaccine. The dosage recommended is in all cases small, and improvement has been observed in several patients to follow the administration of no more than 100 streptococci plus 100 staphylococci: since the stock streptococcal vaccine contains 155 different strains, it would therefore seem that the subcutaneous introduction once a week of, at most, one streptococcus of the strain responsible for the illness might be all that is necessary.

**National Health Insurance.** By G. F. MCCLEARY, M.D. Pp. x., 185. London: H. K. Lewis & Co. Ltd. 1932. Price 6s.—This little book gives a most interesting account of the origin and growth of National Health Insurance in this and other countries. It contrasts the various methods adopted by different states, and discusses very concisely the advantages and disadvantages of their characteristic features, and how well (or otherwise) they work; also their effect on the populace, the medical profession, the Treasury and Friendly Societies. On page 132 is a good story about "Christmas Fever"—a raging epidemic, confined to insured persons, starting in the middle of December and terminating—always favourably—on the Monday after Epiphany. There is a valuable international bibliography.

**The Legal and Ethical Aspects of Medical Quackery.** By L. Le Marchant Minty, Ph.D. Pp. xviii., 262. London: William Heinemann, Ltd. 1932. Price 7s. 6d.—This very engaging volume of some 260 pages, though packed with excellent material, is, on the whole, rather easier to read than to review. Perhaps the first criticism that occurs to one is that the text is happier than the title. Dealing as it does so much with orthodox medicine, it might well have been called "The Law in Relation to Medical Practice," and it could certainly be read with much profit by any recently-qualified man. Here and there, of course, the contents betray the layman, as on page 23, for instance, he infers that chloroform and ether come into the schedules of the Dangerous Drugs Act, and in the first chapter he seems to be unaware that there is a M.R.C.P. (Lond.) as well as the Fellowship and Licentiate. His views that Fellows of the Royal College of Physicians could sue for their fees under some other diploma has the merit of novelty, and I commend it to those of my medical colleagues so qualified who feel themselves handicapped in this way. In conclusion, one may say that this volume breaks new ground, and its wealth of information should make it an important addition to the bookshelves of every practitioner.

**A System of Surgery.** By C. C. Choyce, C.M.G., C.B.E., M.D., F.R.C.S. Third Edition. Three volumes. Illustrated. London: Cassell & Co. Ltd. 1932. Price £6.—Choyce's *System of Surgery* needs no introduction to the profession, with whom it has been deservedly popular since its first publication in 1911. In the present edition fourteen new contributors make their appearance, while some of those previously contributing have dropped out. All the articles have been extensively revised or rewritten in the light of recent knowledge. There is, for example, an entirely new article by Mr. Birkett upon the uses of radium, while those dealing with the surgery of the parathyroids and of the sympathetic system have been brought fully up to date. Mr. Groves provides an excellent chapter upon fractures and Mr. Wilfred Trotter's thoughtful and original contributions dealing with the surgery of brain and cord have been revised by Mr. Julian Taylor.

The printing is as good as ever and numbers of fresh illustrations have been added. Mr. Choyce and his collaborators are to be congratulated upon having carried out a difficult task in a highly satisfactory manner.

**A Short Practice of Surgery.** By HAMILTON BAILEY, F.R.C.S., and R. J. MCNEILL LOVE, M.S. Vol. II. Pp. vii., 531–1005. Illustrated. London: H. K. Lewis & Co. Ltd. 1932. Price 20s.—It is a question whether Bailey and Love, who wrote this second volume of their *Short Practice of Surgery*, or the students who will read it are the more to be envied. Certainly the work is a marvel among medical text-books and represents as great an advance in teaching the subject as modern operative technique does in practical achievement. It covers the surgery of the chest, abdomen, head, central nervous and integumentary system. The best surgical procedures known to-day, whether just out or time honoured, are the only ones included. The same applies to the descriptions and classification of surgical diseases. As a result of this dexterous selection and simplification of the voluminous literature of the day, a student now, for the first time, can well and truly encompass the whole realm of general surgery in the short years which the teaching curriculum allows. For not only are there delightful lucidity of language and pleasing style, but also splendid, memorable illustrations, and beautiful and faultless letterpress. It is a common complaint of the whole wide range of the surgical text-books that, though any one may excel in some features, they fail miserably in others and leave the student dissatisfied. But in this compendium is realized the student's dream of a book that leaves nothing out which he wants to know. More than that, the method of arrangement of the text and the comprehensive lists of affections of different organs make the well-packed information most easily assimilable. No surgical guide contains such encyclopedic information nor displays its contents with such marvellous intelligence.

**The Injection Treatment of Varicose Veins, Hæmorrhoids and other conditions.** By R. H. MAINGOT, F.R.C.S. Pp. ix., 100. London: H. K. Lewis & Co. Ltd. 1932. Price 4s.—On the latest publication on injection treatment Mr. Rodney H. Maingot is to be congratulated. His new book, *The Injection Treatment of Varicose Veins, Hæmorrhoids, and other Conditions*, not only contains the latest but also the best practical information on this expanding field of therapy.

It is an admirable guide to various methods and a safeguard against pitfalls. The need is emphasized for a preliminary general, as well as a local, examination of the patient proposed for injection. The list of contra-indications is particularly valuable for beginners in the treatment. A novel method for dealing with saphena varix in the groin is described, called "twin injection," which is also efficacious for resistant veins. That urticaria may ensue in the use of sodium morrhuate is news to the reviewer, and a valuable warning. The Dickson Wright method of treating varicose ulcers is clearly recounted, and his views on the defective venous hydraulics underlying the condition explained. The two chapters on injections for hæmorrhoids and for obliterating troublesome synovial and serous sacs fully informs readers desirous of giving such methods a trial.

**Diseases of the Nose, Throat and Ear.** Edited by A. LOGAN TURNER, M.D., LL.D. Third Edition. Pp. xxvi., 465. Illustrated. Bristol: John Wright & Sons, Ltd. 1932. Price 20s.—This is easily the best text-book on the subject, both for student and for practitioner, and the third edition maintains the high standard of its forebears. We offer it, therefore, a full and hearty welcome, and it is in no carping spirit that we venture one criticism. The space devoted to major operations is wasted: few of the readers will ever perform one. And there is beginning a tendency even more disastrous in books than in men—with age comes obesity.

**The Principles and Practice of Otology.** By F. W. WATKYN-THOMAS, F.R.C.S., and A. LOWNDES YATES, M.C., F.R.C.S. Pp. xii., 555. Illustrated. London: H. K. Lewis & Co. Ltd. 1932. Price 25s.—This book will be read by all otologists: it is doubtful whether it will appeal to others, and perhaps this is as well, as the authors are inclined to present their own opinions as accepted doctrine. It consists of a series of extremely interesting essays on otological subjects, principally the investigation of types and degrees of deafness and their graphic representation; the pathology of aural disease; and details of operative technique. There is much that is new (including the construction "Guttæ pro auris") or is considered from a new angle, though the authors sometimes claim as their own what has been long established, for example, that the upper limit of audible tones descends with advancing age. Some of the omissions are hard to understand:

skiagraphy is mentioned in the diagnosis of foreign bodies, but not in connection with otitis media ; aural ionisation and parathyroid in the treatment of otosclerosis are both mentioned but not described. To each chapter is appended a valuable bibliography of recent literature. There are 70 plates, well chosen and beautifully reproduced, the great majority from otological and anatomical publications ; over 50 audiographic charts, and nearly 100 diagrams and sketches, of which latter many fail to reach the general standard of excellence. The index is adequate.

**Recent Advances in Pathology.** By G. HADFIELD, M.D., and L. P. GARROD, M.B. Pp. x., 392. Illustrated. London : J. & A. Churchill. 1932. Price 15s.—This addition to the "Recent Advances Series" is especially welcome at the present time, since it affords a ready means of ascertaining the more practical results of pathological research during recent years. Hitherto it has been almost impossible for the clinician to unearth from masses of detail in scattered journals any tangible evidence of pathological opinion on any given subject. The authors have succeeded in a most conspicuous manner in presenting a volume containing a careful and authoritative résumé of pathological research contributing to the better understanding of the causation and effects of disease processes. The first six chapters form an excellent epitome of work accomplished on the reticulo-endothelial system and cancer research, and constitute a well-balanced corrective to the many erroneous views which have acquired undeserved publicity. The cardiovascular system is dealt with concisely, and our knowledge of essential hypertension is carefully scrutinized. Bright's disease and renal œdema have received considerable attention with the result that the pathological and clinical aspects are brought into a more reasoned relationship. Stewart's work on peptic ulceration receives due recognition. The chapter on the ductless glands includes recent discoveries on the relationship of the parathyroids to certain skeletal diseases, and envisages important advances in our knowledge of the supra-renals and the treatment of Addison's disease. Underlying the whole book one can discern a clinical acumen of undoubted quality which cannot fail to enhance academic pathology.

